# Caveolin-1 Expression Ameliorates Nephrotic Damage in a Rabbit Model of Cholesterol-Induced Hypercholesterolemia

**DOI:** 10.1371/journal.pone.0154210

**Published:** 2016-04-28

**Authors:** Ya-Hui Chen, Wei-Wen Lin, Chin-San Liu, Li-Sung Hsu, Yueh-Min Lin, Shih-Li Su

**Affiliations:** 1 Vascular and Genomic Center, Changhua Christian Hospital, Changhua, Taiwan; 2 Institute of Biochemistry and Biotechnology, Chung Shan Medical University, Taichung, Taiwan; 3 Department of Internal Medicine, Division of Cardiovascular Center, Taichung Veterans General Hospital, Taichung, Taiwan; 4 Graduate Institute of Integrative Medicine, China Medical University, Taichung, Taiwan; 5 Department of Pathology, Changhua Christian Hospital, Changhua, Taiwan; 6 Department of Medical Technology, Jen-Teh Junior College of Medicine, Nursing and Management, Miaoli, Taiwan; 7 Department of Internal Medicine, Division of Endocrinology and Metabolism, Changhua Christian Hospital, Changhua, Taiwan; 8 Institute of Medicine, Chung Shan Medical University, Taichung, Taiwan; Beijing Key Laboratory of Diabetes Prevention and Research, CHINA

## Abstract

Caveolin-1 (CAV-1) participates in regulating vesicular transport, signal transduction, tumor progression, and cholesterol homeostasis. In the present study, we tested the hypothesis that CAV-1 improves dyslipidemia, inhibits cyclophilin A (CypA)- mediated ROS production, prevents mitochondrial compensatory action and attenuates oxidative stress responses in cholesterol-induced hypercholesterolemia. To determine the role of CAV-1 in mediating oxidative and antioxidative as well as cholesterol homeostasis, hypercholesterolemic rabbits were intravenously administered antenapedia-CAV-1 (AP-CAV-1) peptide for 2 wk. AP-CAV-1 enhanced CAV-1 expression by ˃15%, inhibited CypA expression by ˃50% (*P* < 0.05) and significantly improved dyslipidemia, thus reducing neutral lipid peroxidation. Moreover, CAV-1 attenuated hypercholesterolemia-induced changes in mitochondrial morphology and biogenesis and preserved mitochondrial respiratory function. In addition, CAV-1 protected against hypercholesterol-induced oxidative stress responses by reducing the degree of oxidative damage and enhancing the expression of antioxidant enzymes. CAV-1 treatment significantly suppressed apoptotic cell death, as evidenced by the reduction in the number of terminal deoxynucleotidyl transferase dUTP nick end-labeling-positive cells. We concluded that CAV-1 plays a critical role in inhibiting CypA-mediated ROS production, improving dyslipidemia, maintaining mitochondrial function, and suppressing oxidative stress responses that are vital for cell survival in hypercholesterol-affected renal organs.

## Introduction

Caveolin-1 (CAV-1) is a cholesterol- and sphingomyelin-binding protein responsible for caveolae formation. It is highly expressed in vascular endothelial cells, adipocytes, smooth muscle cells, and fibroblasts [1]. It also plays an essential role in regulating vesicular transport, signal transduction, tumor progression, and cholesterol homeostasis [2,3]. CAV-1 may mediate cellular cholesterol efflux to high-density lipoprotein (HDL) [4,5]. Several studies have revealed that CAV-1 protein overexpression markedly reduced total cholesterol and free cholesterol (FC) content as well as their accumulation in lipid-loading cells [6–8]. In CAV-1-deficient mice, moderately elevated whole-lung cholesterol content, impaired liver regeneration, cardiovascular disorders, and FC accumulation in mitochondrial membranes resulted in mitochondrial diseases [9–11]. However, Frank *et al*. demonstrated that CAV-1 is proatherogenic in endothelial cells, whereas it is antiatherogenic in smooth muscle cells and macrophages [12,13]. Thus, demonstrating the cell-specific role of CAV-1 during the development of related-disease may be crucial.

Abnormal lipoprotein metabolism causes kidney injuries and contributes to the progression of kidney diseases because of low plasma HDL-C and high oxidized lipid levels [14–16]. Recently, increased lipid influx and reduced reverse cholesterol transport have been reported to cause glomerulosclerosis and tubulointerstitial damage. Cholesterol is considered a crucial regulator in the pathogenesis of kidney diseases [17,18]. In the renal tissue, high CAV-1 expression has been observed in vascular endothelial cells and on distal tubular epithelium membrane surfaces, and high CAV-1 expression in injured proximal tubules may contribute to their regeneration [19,20]. CAV-1 expression in KO mice increases the glomerular filtration rate and transfer of albumin from blood to tissues. Impaired renal calcium reabsorption may lead to hypercalcemia and urolithiasis [21,22].

In this study, antennapedia-conjugated CAV-1 peptide, a drosophila transcription factor facilitating CAV-1 translocation across the cell membrane [23,24], was used in a hypercholesterolemic-rabbit model. Our previous studies have revealed that CAV-1, in association with HDL, can be used to prevent and treat atherosclerosis through the cholesterol efflux pathway. In addition, CAV-1 palliates adverse hepatic reactions in hypercholesterolemic rabbits [25–27]. In the present study, we further investigated the role of AP-CAV-1 in hypercholesterolemia-induced oxidative damage to the kidneys. To evaluate CAV-1-induced changes in the ROS-dependent mechanism in injured renal tissue, we determined the CAV-1, CypA, and neutral lipid distribution; mitochondrial morphology; the biogenesis-mediated protein content; oxidative and antioxidative homeostasis; and apoptosis in the hypercholesterol-affected target organs, the kidneys.

## Materials and Methods

### Experimental protocol

The animal protocols and facilities were reviewed and approved by the Institutional Animal Care and Use Committee of Taichung Veterans General Hospital, Taichung, Taiwan (Permit Number: La-95278). The animals were housed in the animal research facilities of the hospital and were maintained under the care of the facility staff, according to the guidelines of the Animal Ethics committee. All surgery was performed under ketamine and xylazine anesthesia, and all efforts were made to minimize suffering. A total of 18 male New Zealand white rabbits initially weighing approximately 4 kg were divided into three groups. Six of the rabbits that were fed a chow diet (Fu-Shou Co., Taichung, Taiwan) were used as normal diet controls (NCs). The remaining 12 rabbits, which were fed a 2%-cholesterol diet for 8 wk, were used as high-cholesterol diet controls (HCs). Subsequently, at the beginning of the sixth week, six rabbits that were intravenously administered 1 mg·kg^-1^ of AP-CAV-1 peptide [RQPKIWEFPNRRKPWKK-DGIWKASFTTFVTKYWFYR-(OH)] (GU-Yuan Biotech Services Corp., Taiwan) every 2 d for 2 wk were used as high-cholesterol diet with AP-CAV-1 (HCAV-1). The rabbits in each group were sacrificed after 8 wk; they were anesthetized with a mixture of ketamine (40 mg/kg) and xylazine (5mg/kg) given intramuscularly. After cannulation of an ear vein, the animals were sacrificed through intravenous 50 mg/kg ketamine injection. Blood was collected through a cardiac puncture, and the kidneys were dissected for further analysis. Renal DNA was isolated using the GenoMaker reagent kit (Blossom, Taiwan) according to manufacturer recommendations. All animals were monitored at the time of feeding every day during the study periods, and the final sample size was six rabbits per group with a 100% survival rate.

### Analytical measurements

#### Serum biochemistry and kidney tissue analysis

To assess the clinical biochemical changes in the rabbits, the serum concentrations of triglyceride (TG) and free fatty acids (FFAs) were measured using an automatic analyzer (ADVIA 1800; Siemens, Tarrytown, NY, USA). HDL-C and LDL-C were measured using elimination or catalase methods with an automatic analyzer (ADVIA 1800; Siemens) in duplicate. Kidney tissue was embedded in paraffin and sectioned at 4 μm for hematoxylin and eosin (H&E) and examined under an Olympus BX61 microscope (Tokyo, Japan).

#### Real-time PCR

The mitochondrial DNA (mtDNA) copy number in the kidney was measured according to the method of Chen *et al* [27]. PCR amplification was performed using the Platinum^®^ SYBR^®^ Green qPCR SuperMix UDG (Invitrogen, Carlsbad, CA, USA) and examined using an ABI 7700 analyzer (Applied Biosystems, Foster City, CA, USA). Data analysis was based on the cycle threshold (C_T_). The difference in C_T_ values was used as the measure of relative abundance (i.e., C_T_ [mtDNA D-loop]−C_T_ (β-actin)] of the mitochondrial genome.

#### Neutral lipid accumulation and quantification

Fresh renal tissue was immediately frozen in an optimal cutting temperature compound (Sakura Finetek, Torrance, CA, USA), and 7-μm-thick cryostat sections were obtained and stained with 0.2% Oil Red O solution. Nuclei were counterstained with hematoxylin, and the slides were mounted using Clear Mount^TM^ mounting solution (Invitrogen; Eugene, OR, USA) and examined under an Olympus BX61 microscope (Tokyo, Japan). For animal kidney tissue, positive cells were quantified in five fields per animal at 200×, with six rabbits for each group per time point (Nuance inForm analysis).

#### Malondialdehyde measurement

To assess lipid peroxidation in the rabbits, the concentration of malondialdehyde (MDA) in the renal supernatant was measured using an NWLSS^TM^ MDA assay kit (Northwest, Vancouver, WA, USA). Absorbance was measured at 532 nm and expressed as nanomoles per milligram of the renal protein.

#### Electron microscopy

To observe the mitochondrial morphological changes, the kidney slices (1 mm^3^ thick) were manually prepared and immediately placed onto a cold fixative comprising a mixture of 4% formaldehyde and 1% glutaraldehyde in 0.2-M Cacodylate buffer (pH = 7.4) for 6 h, postfixed with osmium tetraoxide, and embedded in Spurr’s resin. Ultrathin sections were double stained with uranyl acetate and lead citrate and examined under a JEM-1230 electron microscope (JEOL Ltd., Japan).

#### 8-OHdG and oxidative mtDNA damage measurement

The expression of 8-hydroxy-2'-deoxyguanosine (8-OHdG) in renal tissue was determined through immunofluorescence staining. The primary antibody used was monoclonal anti-8-OHdG (1:4000; Abcam, Cambridge, MA, USA). An AlexaFluor®488-conjugated antimouse IgG was used as the secondary antibody. Nuclei were stained with Hoechst. The images were quantified using a fluorescence microscope, Olympus BX61 (Tokyo, Japan, Image-Pro Plus 4.5). The degree of oxidative mtDNA damage was defined as △Ct, which was the difference between CtT (the Ct value from the OGG1-treated DNA sample) and CtN (the Ct value from the untreated DNA sample). A higher △Ct resulted in higher 8-OHdG levels and oxidative mtDNA damage, as described by Lin *et al* [28].

#### Immunohistochemistry and quantification

Immunohistochemistry was performed as described previously [27]. The following primary antibodies were used: monoclonal anti-CAV-1 (1:250; Epitomics, Burlingame, CA, USA), monoclonal CyP A (1:150; GeneTex, Irvine, CA, USA), monoclonal anti-peroxisome proliferator-activated receptor-γcoactive 1α (PGC-1α; 1:250; Abcam), polyclonal anti-nuclear respiratory factor-1 (NRF-1; 3 μg/mL; GeneTex), polyclonal anti-superoxide dismutase 2 (SOD2; 1:1000, Novus, Littleton, CO, USA), and polyclonal anti-catalase (1:1000; Abcam). monoclonal anti-nuclear factor E2-related factor 2 (Nrf2; 1:250; GeneTex), polyclonal anti-Kelch-like ECH- associated protein 1 (Keap1; 1:150; Bioss, Woburn, MA, USA), and polyclonal anti- glutamate-cysteine ligase catalytic subunit (GCLC; 1:100; GeneTex). Nuclei were counterstained with hematoxylin and photographed using an Olympus BX61 microscope (Tokyo, Japan). For animal kidney tissue, positive cells were quantified in five fields per animal at 400×, with six rabbits for each group per time point (Image-Pro Plus 4.5).

#### Immunoblot analysis

Tissue lysate was harvested in a lysis buffer (25 mM bicine, 150 mM sodium chloride, pH 7.6; Pierce), homogenized, and centrifuged for 20 min at 12 000 rpm at 4°C. Nuclear and cytoplasmic fractions were prepared from renal tissue using a Nuclear Protein Isolation-Translocation Assay Kit (FIVEphoton Biochemicals, San Diego, CA, USA) according to manufacturer instructions. The protein concentration was detected using a BCA protein assay kit (Pierce BCA assay, Thermo Scientific, Rockford, IL). Proteins (30 or 50 μg) were separated using 8% –12% SDS-PAGE and then transferred to PVDF membranes (Pall Corporation). Blots were then probed with monoclonal anti-CAV-1 (1:1000; Epitomics), monoclonal Cyp A (1:1000; GeneTex), monoclonal anti-PGC-1α (1 μg/mL; Abcam), polyclonal anti-NRF-1 (1:1000; GeneTex), polyclonal anti-SOD2 (1:1000, Novus), polyclonal anti-catalase (1:1000; Abcam), MitoProfile Total OXPHOS rodent antibody cocktail (1:800; MitoSciences, Eugene, OR), monoclonal anti-Nrf2 (1:800; GeneTex), polyclonal anti-Keap1 (1:1000; Bioss), polyclonal anti-GCLC (1:800; GeneTex), polyclonal anti-Lamin B1 (1:1000; Abcam), mouse anti-GAPDH (1:1000; Abcam), and mouse anti-β-actin (1:10000; Millipore, Billerica, MA, USA). Signals were obtained using an enhanced chemiluminescence kit (Millipore), and densitometry was performed using Fusion-Capt software (Vilber Lourmat, Fusion FX7, France).

#### Apoptosis

Four-micrometer-thick renal sections were deparaffinized with xylene, rehydrated in ethanol, and washed with a PBS buffer. The sections were incubated with proteinase K (25 μg/mL) for 30 min at room temperature (RT) to expose DNA for end labeling. A positive control renal section was exposed to a DNase I solution for 30 min at RT. After incubation, cell death was detected in renal sections by using a terminal deoxynucleotidyl transferase dUTP nick end-labeling (TUNEL) assay kit (Invitrogen) according to manufacturer instructions. The sections were examined using fluorescence microscopy under an Olympus BX61 microscope. TUNEL-positive cells were quantified in seven fields per animal at 400×, in six rabbits for each group per time point (Image-Pro Plus 4.5).

### Statistical analysis

Statistical analyses were performed using one-way ANOVA and *t* tests. Data are presented as means ± SD from three independent experiments; *P* < 0.05 was considered statistically significant.

## Results

### CAV-1 improves serum LDL-C, TG, and FFA concentrations

To determine whether CAV-1 increased the circulating cholesterol and lipid efflux in hypercholesterolemic rabbits (the high-cholesterol diet control group), we measured serum TG, HDL-C, LDL-C, and FFA levels. CAV-1 significantly lowered serum TG, LDL-C, and FFA levels in the HCAV-1 group compared with the age-matched HC group (52.0 ± 4.72 vs. 72.33 ± 13.53 mg/dL, 211.7 ± 85.1 vs. 248.7 ± 9.8 mg/dL, and 0.33 ± 0.02 vs. 0.40 ± 0.10 mmol/L, respectively, Table 1). However, no significant difference was observed for serum HDL-C levels in the HCAV-1 and HC groups. CAV-1 significantly affected these clinical biochemical indices.

**Table 1:** Serum biochemical data in experimental animals.^1^

Group	TG mg/dL	HDL-C mg/dL	LDL-C mg/dL	FFA mmol/L
**NC**	40.67± 3.18	23.33 ± 4.33	9.67 ± 0.33	0.19 ± 0.01
**HC**	72.33 ±13.53	27.00 ± 0.58	248.7 ± 9.8*	0.40 ± 0.10
**HCAV-1**	52.00 ± 4.72	26.33 ± 3.76	211.7 ± 85.1	0.33 ± 0.02
***P*-value**	0.095	0.722	0.029	0.102

^1^Values are means ± SEM, *n* = 6 in each group. NC group: normal diet without any treatment; HC group: fed 2% cholesterol diet; HCAV-1 group: fed 2% cholesterol diet and treated with caveolin-1 peptide. Values in HC groups with symbol

* represent significant difference from that of the NC group at *P* < 0.05 by one-way ANOVA with least significant difference. TG, triglyceride; HDL-C, high-density lipoprotein cholesterol; LDL-C, low-density lipoprotein cholesterol; FFA, free fatty acid.

### Increased CAV-1distribution, reduced neutral lipid peroxidation, and protected tubular nephrocalcinosis and pyelonephritis

**Fig 1:**
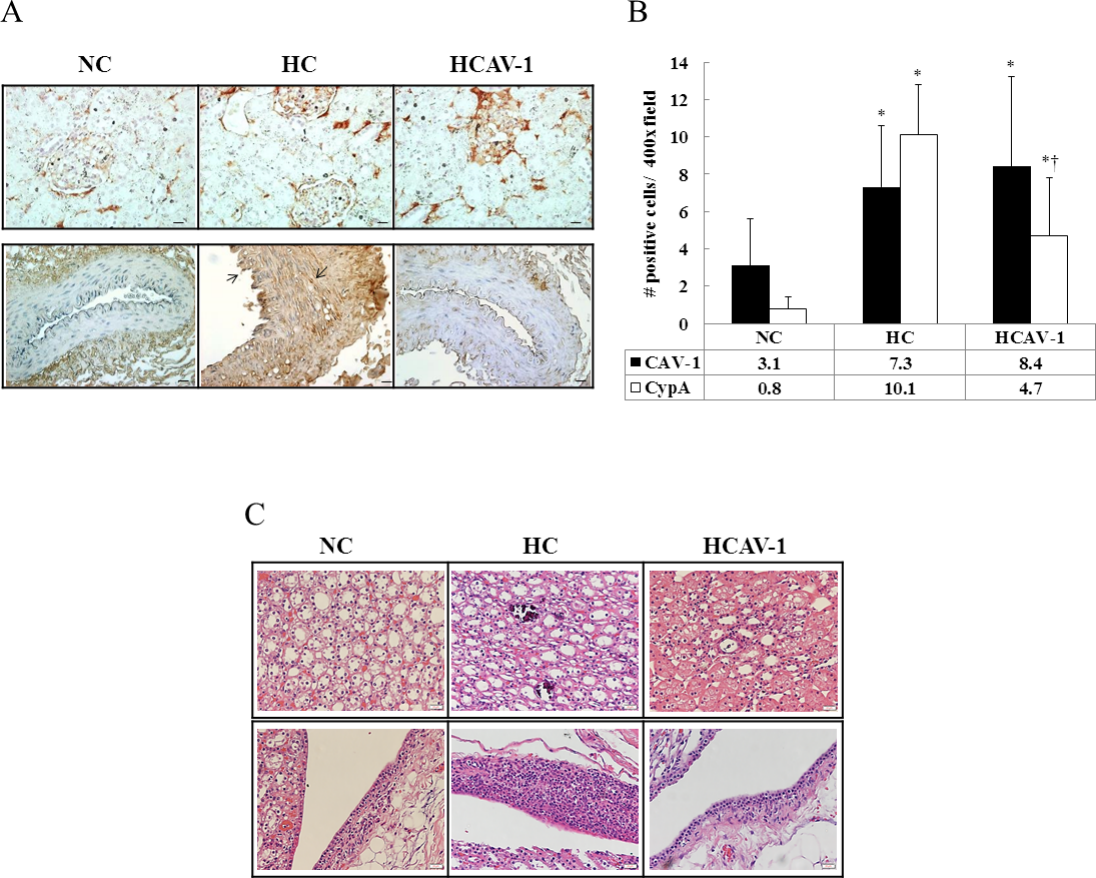
CAV-1 reduces inflammatory activity and then protects tubular nephrocalcinosis and pyelonephritis. (A) CAV-1 is well-distributed in the glomerulus (*upper*). Staining intensity was higher in the AP-CAV-1-treated rabbits (the HCAV-1 group) than in the untreated hypercholesterolemic rabbits (the HC group). CypA was expressed in the endothelial and smooth muscle cells of the renal vasculature (*lower*, arrows). CypA was significantly induced in the HC group. (B) Quantification of CAV-1 and CypA on immunohistochemical staining in rabbit renal tissue per 400× field for five fields per animal; *n* = 6 rabbits for each group, scale bar = 1 μm. (C) H&E-stained renal sections from extensive calcium salt in and around the renal tubules, and the inflammatory cell-infiltrated HC group (*middle*, magnification 400×, bar = 20 μm). The data represent at least three independent experiments; * and † differ significantly for the NC and HC groups, respectively, at *P* < 0.05.

**Fig 2:**
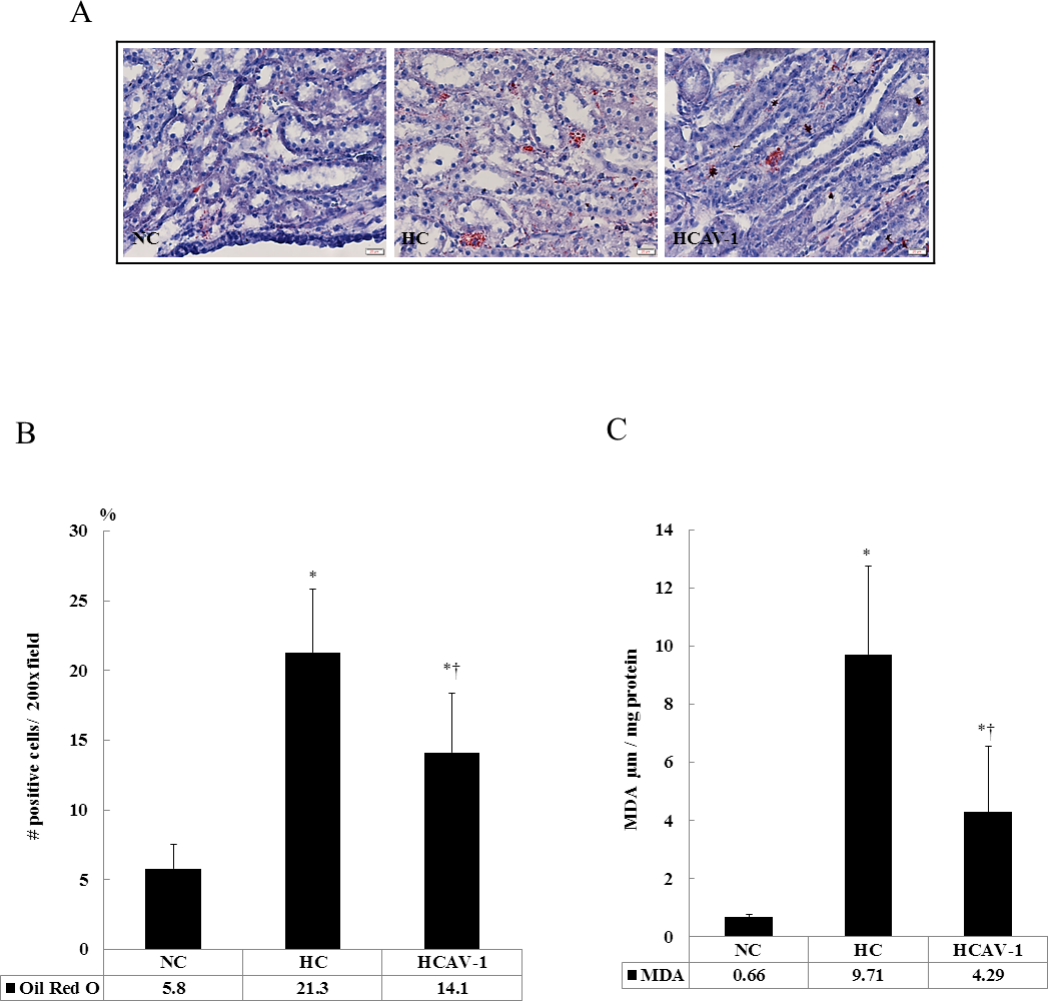
CAV-1 facilitates decreased lipid accumulation for reducing lipid peroxidative production. (A) Neutral lipid accumulation in the renal sections, indicated through Oil Red O staining, was significantly higher in the HC group than in the HCAV-1 group (magnification 400×, bar = 20 μm). (B) Quantification of Oil Red O staining in rabbit renal tissue per 200× field for five fields per animal; *n* = 6 rabbits for each group. (C) A high-cholesterol diet significantly increased MDA levels in renal tissue. The data represent at least three independent experiments; * and † differ significantly for the NC and HC groups, respectively, at *P* < 0.05.

Based on renal section analysis, the staining intensity of CAV-1 was higher in the AP-CAV-1-treated HCAV-1 group than in the HC group and NC rabbits. CAV-1 was chiefly localized in the glomerulus (8.4 ± 4.8 vs. 7.3 ± 3.3 vs. 3.1 ± 2.5 positive cells/400× field, *P* ˂ 0.05). Moreover, AP-CAV-1 markedly reduced the number of CypA-positive cells, which induce inflammation, in the AP-CAV-1-treated HCAV-1 group compared with the HC group (4.7 ± 3.1 vs. 10.1 ± 2.7 positive cells/400× field, *P* ˂ 0.05, Fig 1A and 1B and Dataset in S1 File). H&E-stained renal section from extensive calcium salt in and around the renal tubules, and inflammatory cell-infiltrated HC group (middle, Fig 1C). The renal section in the control group contained markedly more Oil Red O-positive cells, indicating neutral lipid accumulation, than the NC and HCAV-1 groups did (21.3 ± 4.5 vs. 5.8 ± 1.7 vs. 14.1 ± 4.3 positive cells/200× field, *P* ˂ 0.05, Fig 2A and 2B and Dataset A in S2 File). Significantly high MDA levels were observed in the HC group compared with the HCAV-1 and NC groups (9.71 ± 3.03 vs. 4.29 ± 2.27 vs. 0.66 ± 0.10 μM/mg protein, *P* ˂ 0.05, Fig 2C and Dataset B in S2 File). These findings indicate that CAV-1 treatment reduced lipid accumulation, reducing lipid peroxidative production and the inflammatory activity of vascular cells, and protected tubular nephrocalcinosis and pyelonephritis in the hypercholesterol-affected target organs, the kidneys.

### CAV-1 protects mitochondrial morphology and prevents mitochondrial compensatory action

**Fig 3:**
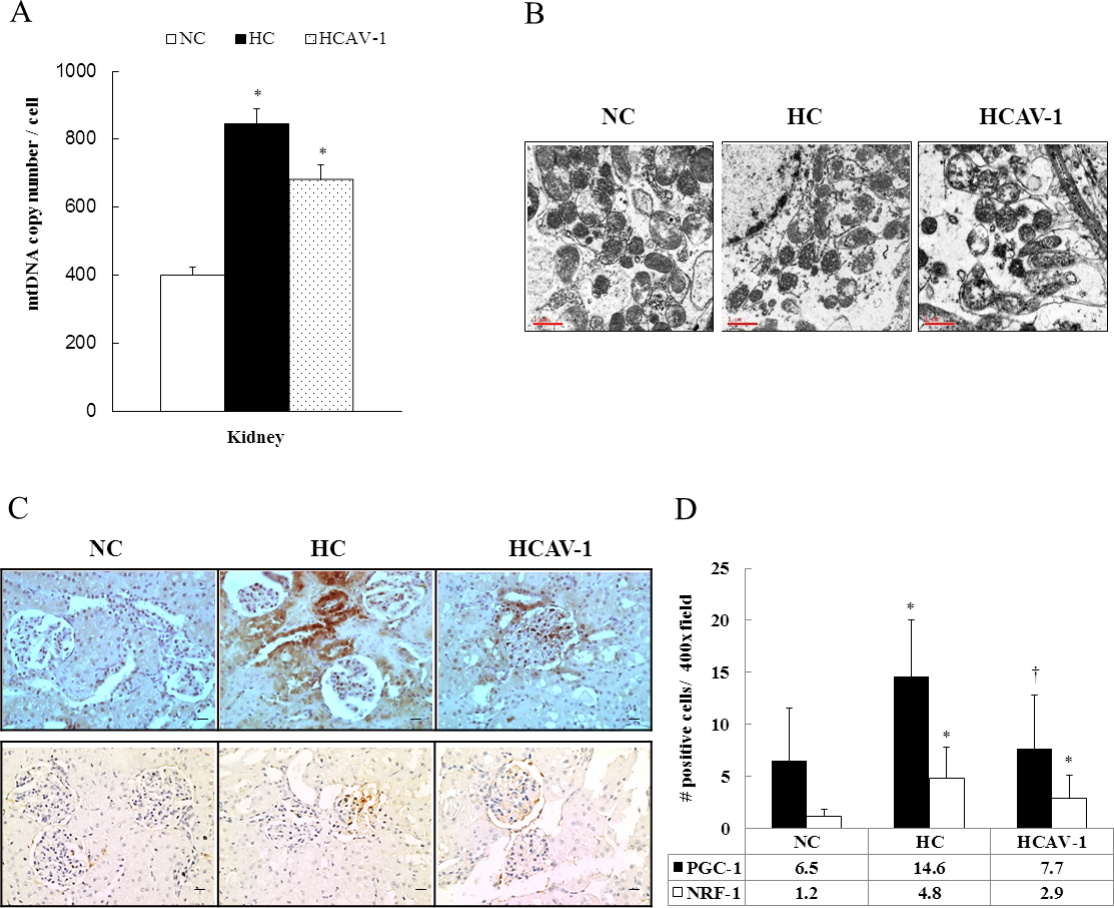
CAV-1 preserves mitochondrial morphology and reduces mitochondrial biogenesis. (A) A significantly high mtDNA copy number per cell was observed in the HC group than in the NC and HCAV-1 groups. (B) Electron microscopy revealed a darker matrix and integrated mitochondrial membranes after CAV-1 treatment (magnification 20000×, *n* = 6 for each group, bar = 1.0 μm). (C) Expression levels of PGC-1α (*upper*) and NRF-1 (*lower*) proteins in mitochondrial biogenesis were markedly low in the CAV-1-treated HCAV-1 group compared with the untreated HC group (magnification 400×, bar = 1 μm). (D) Quantification of PGC-1α and NRF-1 on immunohistochemical staining per 400× field for five fields per animal, *n* = 6 rabbits for each group. The data represent at least three independent experiments; * and † differ significantly for the NC and HC groups, respectively, at *P* < 0.05.

To determine the effects of CAV-1 on mitochondrial differentiation and morphology, the AP-CAV-1-treated hypercholesterolemic rabbits were analyzed using real-time PCR, electron microscopy, and immunohistochemical assays. The real-time PCR assay results revealed that the mtDNA copy number per cell was significantly lower in the HCAV-1 group than in the HC group (Fig 3A and Dataset A in S3 File). Electron microscopy revealed markedly dispersed mitochondria and the presence of intramitochondrial residual bodies or the loss of integrity of the mitochondrial membranes in the hypercholesterolemic rabbits compared with the normal rabbits. In the AP-CAV-1 treated rabbits, CAV-1 protected the hypercholesterolemia-associated and altered mitochondrial morphology, as demonstrated by the dark matrix and integrated mitochondrial membranes (Fig 3B). In addition, immunohistochemistry revealed significantly higher PGC-1α and NRF-1 protein expression in the HC group than in the NC group. However, the PGC-1α and NRF-1 expression levels notably decreased by 1.9- and 1.7-fold, respectively, in the AP-CAV-1 treated group compared with the HC group (*P* ˂ 0.05, Fig 3C and 3D and Dataset B in S3 File). Overall, our results revealed that CAV-1 diminished renal mitochondrial injuries, preserved the mitochondrial morphology, and prevented mitochondrial compensatory action from becoming excessively active in the CAV-1-treated hypercholesterolemic rabbits.

### CAV-1 reduces the degree of oxidative damage

**Fig 4:**
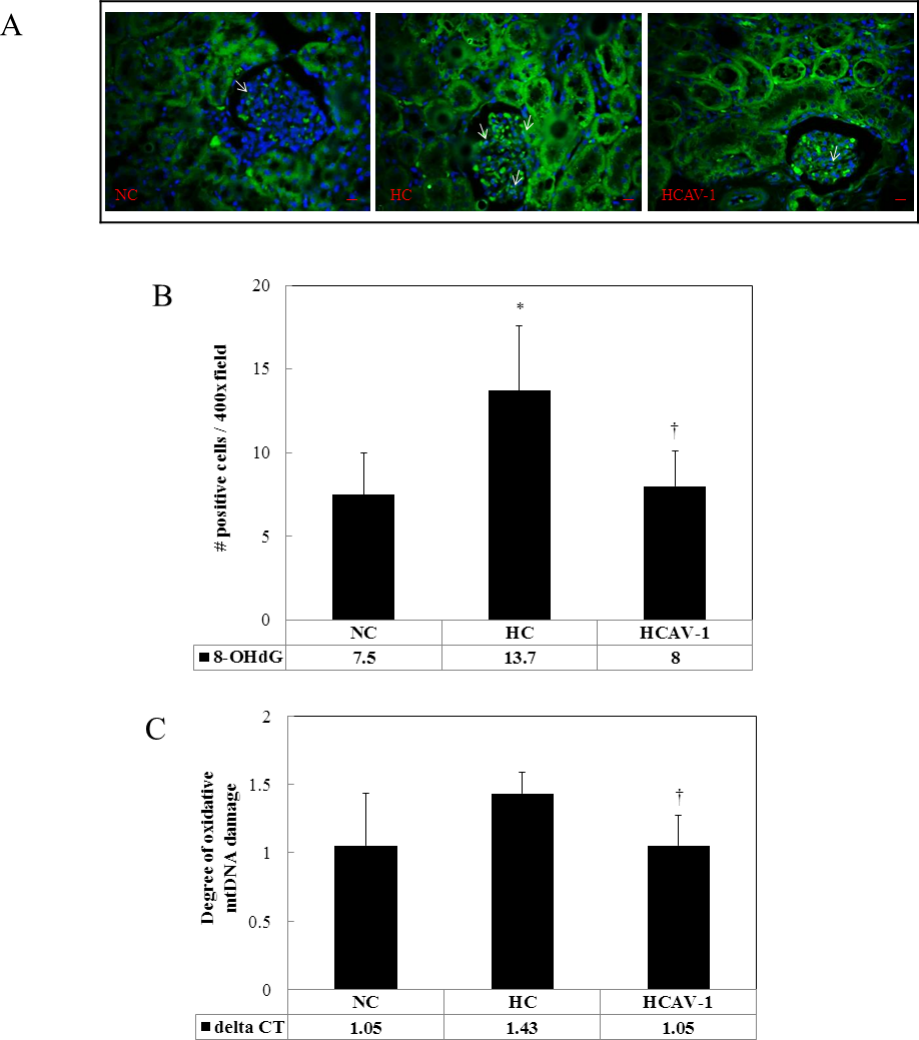
CAV-1 reduced the degree of oxidative damage. (A) 8-OHdG-positive cell signals (arrows) were upregulated more for the rabbits in the HC group (high-cholesterol diet) than for those in the NC and HCAV-1 groups. (B) Quantification of 8-OHdG IHF staining in rabbit glomerular tissue per 400× field for four fields per animal, with six rabbits for each group. (C) Similarly, the degree of oxidative DNA damage in mtDNA was significantly high in the HC group. The data represent at least three independent experiments; * and † differ significantly for the NC and HC groups, respectively, at *P* < 0.05 (magnification 400×, bar = 1 μm).

To determine whether AP-CAV-1 treatment was associated with local reduction of oxidative stress, we measured the 8-OHdG levels and degree of mtDNA damage in renal tissue. A significant reduction was observed for both markers in the AP-CAV-1-treated rabbits. The 8-OHdG expression levels in the glomeruli were significantly lower in the HCAV-1 group than in the HC group (8.0 ± 2.1 vs. 13.7 ± 3.9 positive cells/400× field, *P* < 0.05, Fig 4A and 4B and Dataset A in S4 File). The mtDNA damage (△Ct) values in the kidneys decreased notably in the AP-CAV-1-treated rabbits compared with the HCs (1.05 ± 0.22 vs. 1.43 ± 0.16, *P* < 0.05, Fig 4C and Dataset B in S4 File). In addition, the 8-OHdG expression levels and degree of mtDNA damage were similar in the HCAV-1 and NC groups (7.5 ± 2.5 positive cells/400× field, 1.05 ± 0.38, respectively). These findings suggest that CAV-1 may significantly prevent hypercholesterol-induced oxidative stress, thus reducing oxidative damage in the DNA and mtDNA of renal tissues.

### CAV-1 enhances SOD2 and catalase protein expression

**Fig 5:**
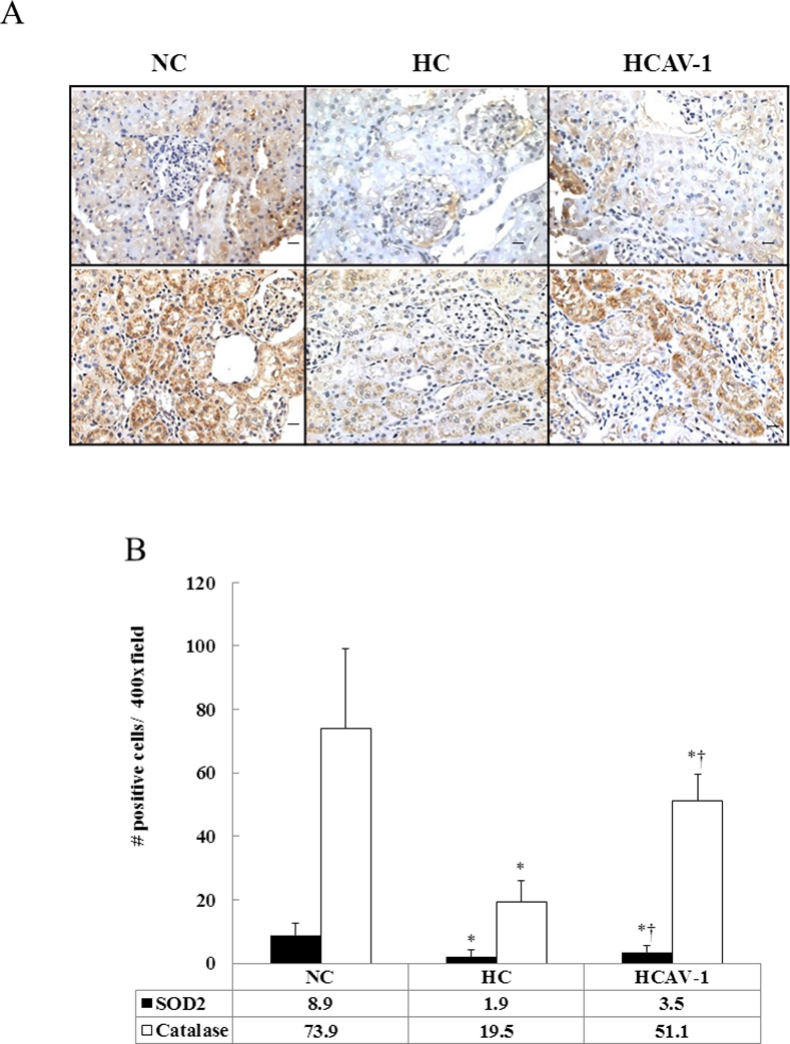
CAV-1 enhances SOD2 and catalase protein expressions. (A) Significantly increased expression of mitochondrial antioxidant enzymes SOD2 (*upper*) and catalase (*lower*) were observed in the CAV-1-treated hypercholesterolemic rabbits. (B) Quantification of SOD2 and catalase. The data represent at least three independent experiments; * and † differ significantly for the NC and HC groups, respectively, at *P* < 0.05 (magnification 400×, bar = 1 μm).

We examined whether AP-CAV-1 treatment exerted additional effects on lowering the expression levels of antioxidant enzymes, such as SOD2 and catalase, in the hypercholesterolemic rabbits. The SOD2 levels were 1.8-fold higher in the HCAV-1 group than in the HC group. Similarly, AP-CAV-1 treatment resulted in a 2.6-fold elevation in the catalase expression (51.1 ± 8.5 vs. 19.5 ± 6.5 positive cells/400× field, *P* ˂ 0.05, Fig 5A and 5B and Dataset in S5 File). Thus, AP-CAV-1 may have improved and attenuated local oxidative stress in the CAV-1-treated hypercholesterolemic rabbits.

### CAV-1 preserves mitochondrial respiratory function by regulating OXPHOS expression

**Fig 6:**
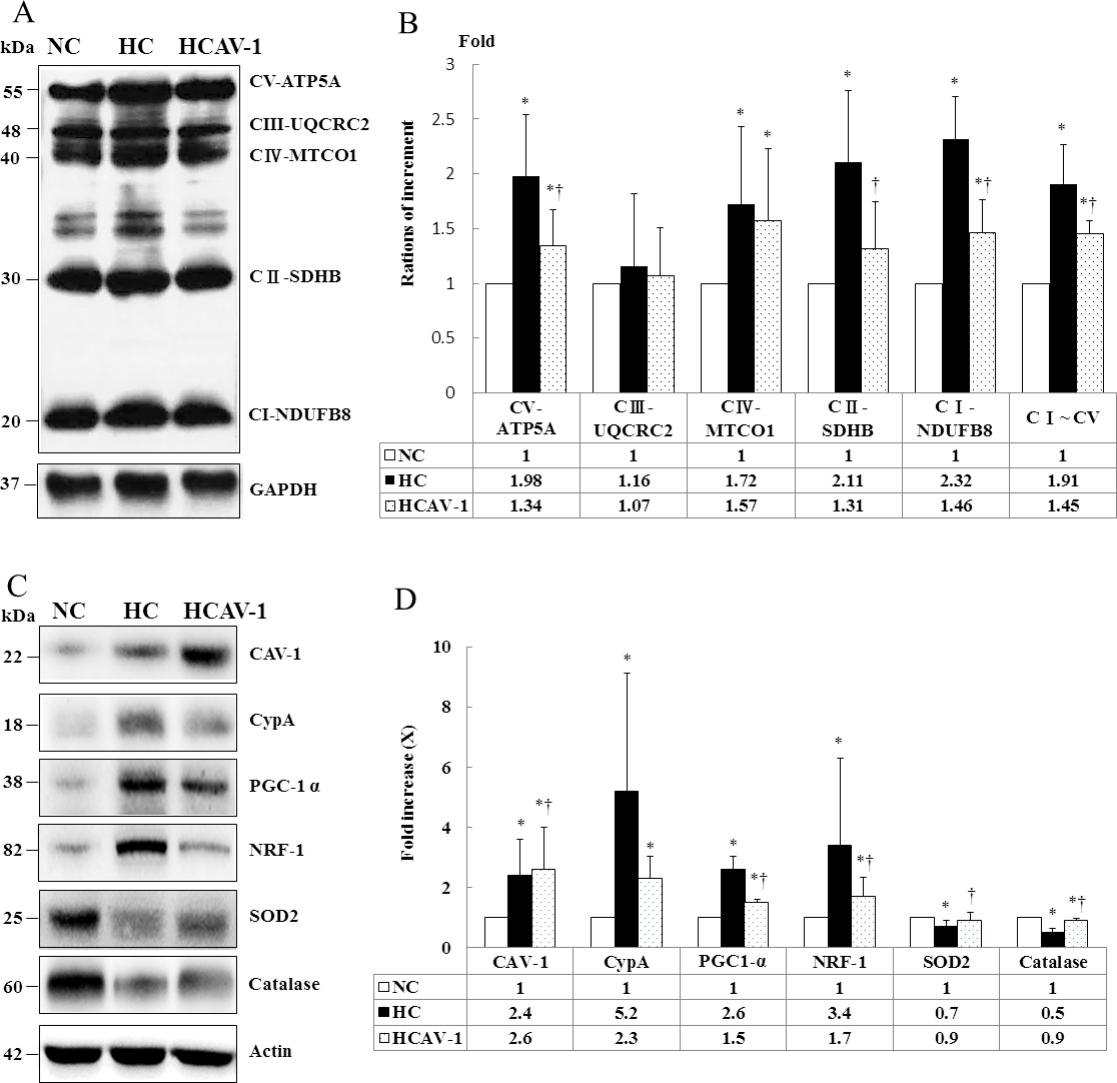
CAV-1 preserves mitochondrial respiratory function through regulation of OXPHOS expression. (A) OXPHOS complex subunits were detected through western blotting with appropriate antibodies. CAV-1 treatment significantly restored hypercholesterolemia-associated increased ETC complex I–V proteins levels. (B) Percentage of the OXPHOS complex (I–V) band intensities is presented in the graph. (C) Representative immunoblot displaying levels of CAV-1, CyPA, mitochondrial biogenesis markers, and antioxidant enzymes in the NC, HC, and HCAV-1 groups. (D) Columns represent average values over three independent experiments. The density for the NC group was set at 1; * and † are significantly different for the NC and HC groups, respectively, at *P* < 0.05. β-actin was used as a loading control. Values are means ± SD.

We used immunoblot analysis for measuring mitochondrial OXPHOS complexes, because they directly affect mitochondrial function and antioxidative capacity. The expression levels of ATP synthase α-subunit (complex V, +0.98-fold), core 2 protein (complex III, +0.16-fold), MTCO1 (complex IV, +0.72-fold), SDHB (complex II, +1.11-fold), and NDUFB8 (complex I, +1.32-fold) were significantly higher in the HC group than in the NC group (*P* < 0.05). In addition, the AP-CAV-1-treated group exhibited a 0.68-, 0.62-, and 0.63-fold reduction in the levels of ATP synthase-α subunit, SDHB, and NDUFB8, respectively, compared with the HC group (*P* < 0.05). Therefore, AP-CAV-1 treatment resulted in significantly lower expression of electron transport chain (ETC) complex I–V proteins (−0.76-fold, *P* < 0.05), suggesting that CAV-1 treatment reserves mitochondrial respiratory function in animals with hypercholesterolemia (Fig 6A and 6B and Dataset A in S6 File). Similarly, the expression of proteins related to mitochondrial biogenesis (PGC-1α, −0.58-fold; NRF-1, −0.50-fold), proinflammation (CypA, −0.44-fold), and antioxidation (SOD2, +1.29-fold; CAT, +1.80-fold) were significantly lower and higher in the AP-CAV-1-treated and HC groups, respectively (*P* < 0.05). CAV-1 protein expression was higher in the CAV-1-treated hypercholesterolemic rabbits (+1.08-fold, *P* < 0.05, Fig 6C and 6D and Dataset B in S6 File). These results were consistent with those obtained in the immunohistochemistry analysis.

### CAV-1 prevented apoptotic cell death

**Fig 7:**
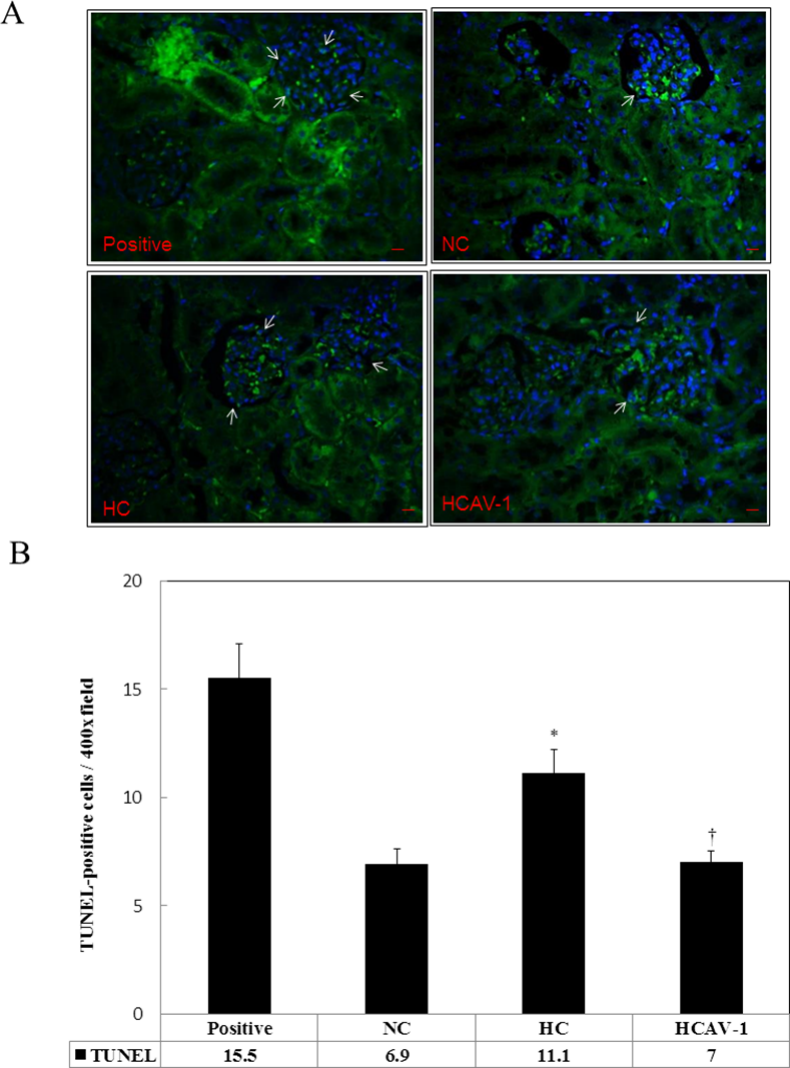
CAV-1 prevents apoptotic cell death. (A) Signals of terminal deoxynucleotidyl TUNEL-positive cells (arrows) were higher in the HC group than in the NC and HCAV-1 groups. Green fluorescence was observed during TUNEL staining (Alexa Fluror 488 nm), and the nuclei were stained with Hoechst dye (blue). (B) Quantification of the TUNEL IHF stain in rabbit renal tissue per 400× fields for seven fields per animal, with six rabbits for each group. A significantly low number of TUNEL-positive cells were observed in the HCAV-1 group; * and † differ significantly for the NC and HC groups, respectively, at *P* < 0.01. Values are mean ± SD from at least three independent experiments.

To investigate whether the CAV-1 activity affected cell survival, we examined apoptotic cell death in glomerular cells by using TUNEL assays. A significant number of TUNEL-positive cells were observed in the control rabbits that were fed a high-cholesterol diet, and the effects were highly significant compared with those in the normal group (11.1 ± 1.1 vs. 6.9 ± 0.7 positive cells/400× field, *P* < 0.05). CAV-1 treatment significantly suppressed apoptotic cell death (7.0 ± 0.5 positive cells/400× field, *P* < 0.05). The imaging and quantification results are summarized in Fig 7A and 7B and Dataset in S7 File. These results suggested that CAV-1 may prevent apoptotic cell death in kidney tissue.

### CAV-1 enhances the Nrf2-mediated antioxidant defense system

**Fig 8:**
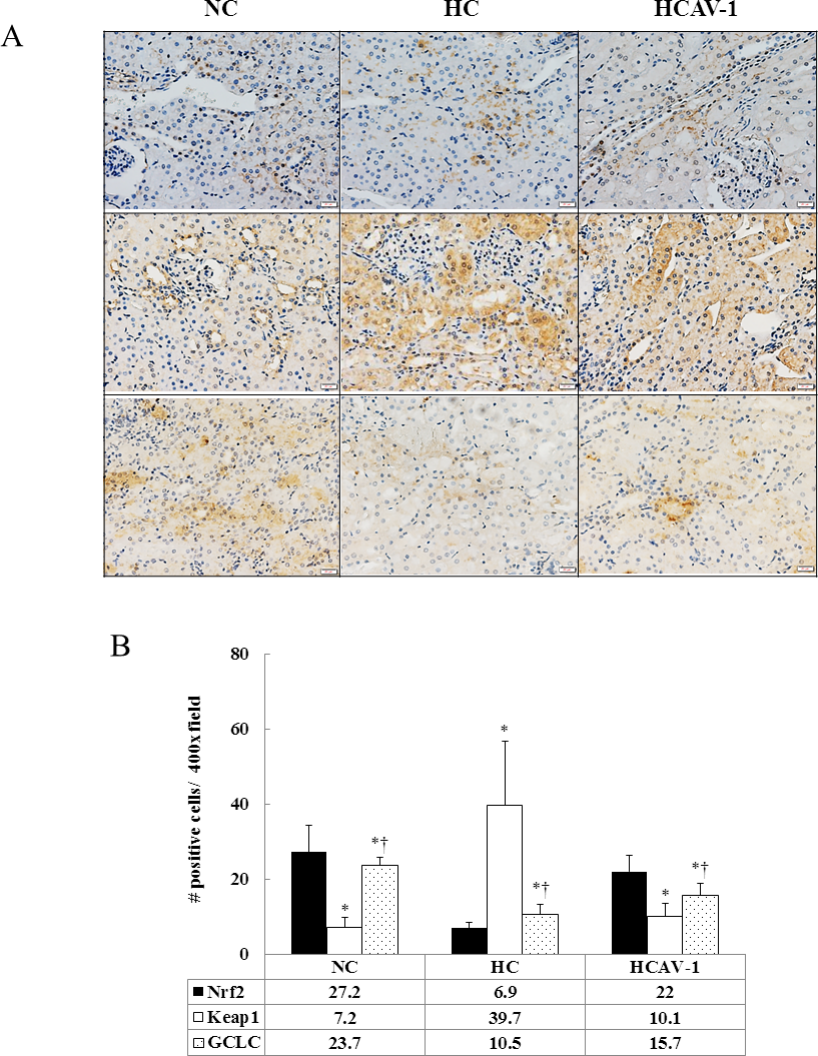
CAV-1 enhances the Nrf2-mediated antioxidant defense system. (A) Staining intensity increased nuclear expression of Nfr2 (*upper*), reduced cytosolic expression of keap1 (*middle*), and increased cytosolic expression of GCLC (*lower*) significantly more in the HCAV-1 group than in the HC group. (B) Quantification of Nrf2, Keap1, and GCLC. The data represent at least three independent experiments; * and † differ significantly for the NC and HC groups, respectively, at *P* < 0.05 (magnification 400×, bar = 20 μm).

**Fig 9:**
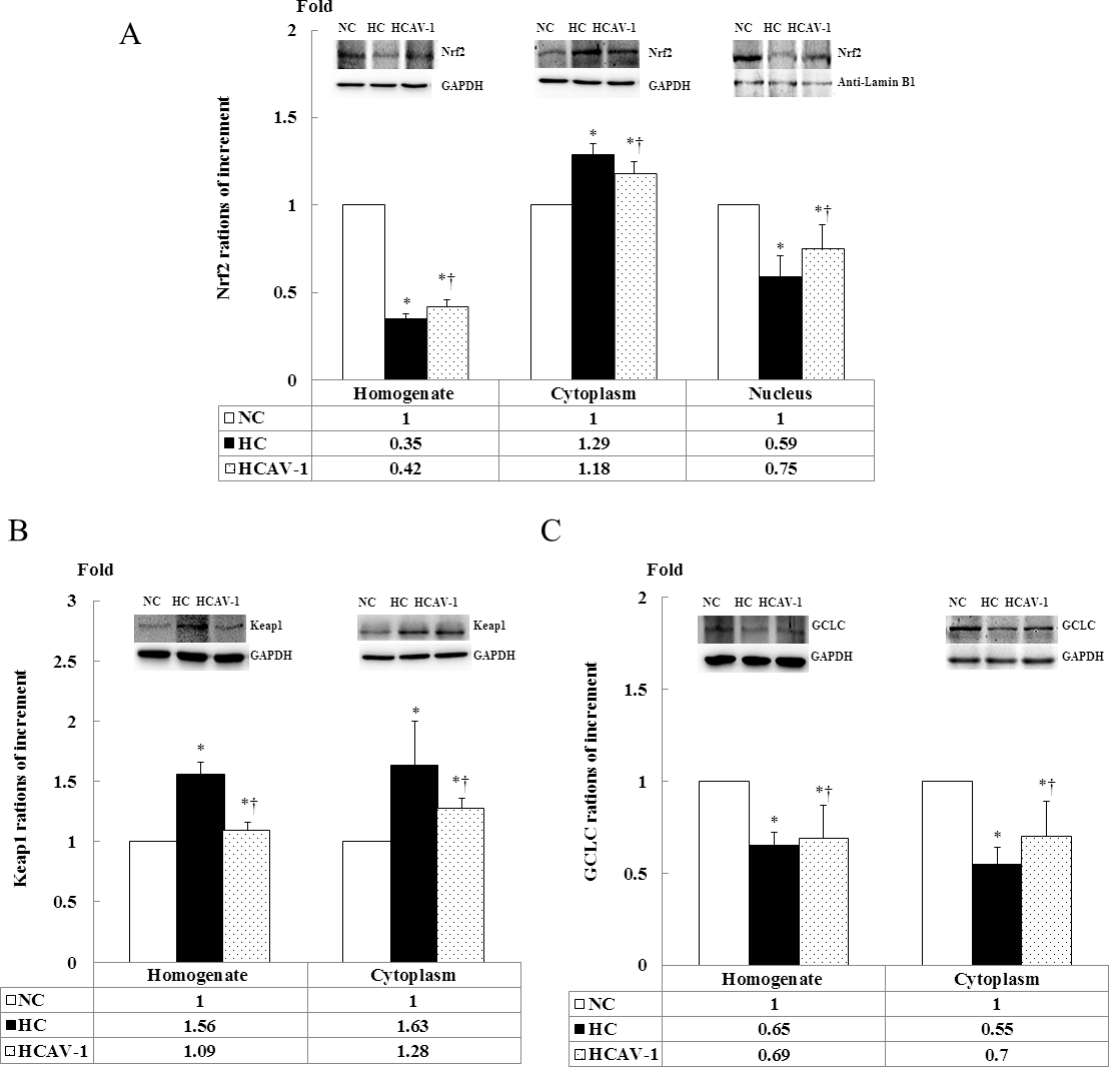
CAV-1 prevents cholesterol impairment in the Nrf2-Keap1-GCLC pathway. (A) Protein expression of Nrf2 was determined in the total homogenate, cytoplasm, and nuclear fractions through western blotting by using GAPDH and anti-Lamin B1 as loading controls. (B) Protein expression of Keap1 was determined in the total homogenate and cytoplasmic fraction through western blotting by using GAPDH as a loading control. (C) Protein expression of GCLC was determined in the total homogenate and cytoplasmic fraction through western blotting by using GAPDH as a loading control. Values from normal rabbit kidney tissue are considered 1 for protein expression. * and † differ significantly for the NC and HC groups, respectively, at *P* < 0.05. Values are mean ± SD from at least three independent experiments.

To further confirm the role of CAV-1 in the antioxidation of cholesterol-induced hypercholesterolemia, for the Nrf2-Keap1-GCLC pathway, the subcellular localization of Nrf2, Keap1, and GCLC was quantified in isolated renal tissue through immunohistochemistry and immunoblot analysis. AP-CAV-1 treatment for the hypercholesterolemic rabbits was significantly increased nuclear expression of Nfr2 by 3.2-fold, the cytosolic keap1 expression was reduced by 75%, and cytosolic GCLC expression was increased by 1.5-fold compared with the HCs (*P* < 0.05, Fig 8A and 8B and Dataset in S8 File). The Nrf2 cytoplasmic protein expression was significantly increased in the renal tissue in the HC group compared with in the NC and HCAV-1 groups, but the levels of Nrf2 in the total protein expression were reduced by 65% and 8% compared with the values obtained from the NC and HCAV-1 groups. Consistent with a decrease in nuclear Nrf2 was also subnormal in the hypercholesterolemic rabbits (*P* < 0.05, Fig 9A and Dataset A in S9 File). Although Keap1 was increased by over 2-fold in the homogenate and cytoplasmic fraction of the hypercholesterolemic rabbit renal tissue compared with that of the normal and CAV-1-treated rabbits (*P* < 0.05, Fig 9B and Dataset B in S9 File). The cholesterol- induced decrease in Nrf2 accumulation in the nucleus was accompanied by decreased homogenate and cytoplasmic fraction GCLC expression. By contrast, treatment with CAV-1 facilitated Nrf2 movement into the nucleus and also prevented a decrease in GCLC expression induced by high cholesterol insult (*P* < 0.05, Fig 9C and Dataset C in S9 File). Overall, these results showed that Nrf2 was mainly in the nucleus and that CAV-1 prevented cholesterol-induced decreases in nuclear Nrf2 expression and GCLC gene transcription as well as enhanced the Nrf2-mediated antioxidant defense system during the development of hypercholesterolemic rabbits.

## Discussion

Using a hypercholesterolemic rabbit model, we have previously demonstrated that CAV-1, in association with HDL-C, can be used not only to prevent but also to treat atherosclerosis through the cholesterol efflux pathway, subsequently palliating adverse hepatic reactions in hypercholesterolemic rabbits [25–27]. In the present study, we investigated the role of CAV-1 in hypercholesterolemia-induced oxidative damage to the kidneys. Our results reveal that (1) CAV-1 can inhibit CypA expression and improve dyslipidemia to reduce neutral lipid peroxidation. (2) CAV-1 attenuates hypercholesterolemia-induced altered mitochondrial morphology and biogenesis and reserves mitochondrial function. (3) CAV-1 provides protection against a hypercholesterol-induced oxidative stress response by reducing the degree of oxidative damage and enhancing the activity of antioxidant enzymes. (4) CAV-1 treatment significantly suppresses apoptotic cell death, as indicated by the reduction in the number of TUNEL-positive cells.

Hypercholesterolemia leads to the development and progression of atherosclerosis and cardiovascular diseases; thus, excluding excess cholesterol from cells and tissue can prevent the occurrence of these diseases [6,7]. In our study, the intracellular TG, LDL-C, and FFA levels increased by 50% when the rabbits were treated with a 2% high-cholesterol diet for 8 wk, whereas the levels decreased substantially when the rabbits were treated with AP-CAV-1. Our findings clearly demonstrate that CAV-1 effectively suppressed intracellular cholesterol and lipid accumulation. CAV-1 reduced cholesterol accumulation *in vivo* and *in vitro* [29,30]. Therefore, our findings are consistent with those of other studies.

Hypercholesterolemia induces proteinuria and increases renal superoxide production and lipid peroxidation, thus lowering renal NO availability. Therefore, renal cortical and glomerular CAV-1 protein expression has been upregulated in rats fed a 2% cholesterol diet for 2 wk [31–33]. These results are similar to those of our study; however, in the rabbits that were fed a 2%-cholesterol diet for a prolonged duration, serum and thoracic aorta CAV-1 protein levels and NO production gradually decreased after 5 wk [25]. Thus, short-term exposure to cholesterol may cause CAV-1 proteins to increase the cholesterol efflux and NO production, and the CAV-1-derived NO may react with superoxides to reduce the lipid peroxidation, thus preventing renal injury. However, in long-term hypercholesterolemia, low renal CAV-1 expression and high renal superoxide activity were observed. Therefore, CAV-1 may exert antioxidant effects by enhancing NO expression in hypercholesterolemia.

Mitochondrial dysfunction is gaining recognition and has been increasingly researched in relation to various diseases. The kidneys are rich in mitochondria and are thus vital energy-generating organs. Therefore, mitochondrial dysfunction plays a crucial role in the pathogenesis of kidney diseases. In kidney diseases, a high amount of FFAs are transferred into the mitochondria, resulting in ATP production, accelerating ROS production, and thus inducing renal cell apoptosis. Furthermore, upregulated COX I and IV expression likely inactivates COX IV expression in the peripheral mononuclear cells of CKD patients at stages 4–5 [34,35]. Oxidative stress has been accompanied by an increase in mitochondrial biogenesis, with PGC-1α regulating cellular bioenergetics in the kidneys after injury [36,37]. Our observations are consistent with those of previous studies: Increased CAV-1 expression not only promotes SOD2, catalase, and Nrf2-GCLC expression and oxidative stress-derived ROS scavenging but also downregulates COX I, IV, and V expression, thus preventing glomerular cell death. In addition, CAV-1 controls mitochondrial biogenesis, thereby reducing PGC-1α and NRF-1 expression. Thus, CAV-1 expression and mitochondrial function are closely associated with renal injury progression.

Previous studies have illustrated that CAV-1 may be involved at the earlies stages of atherosclerosis development through increased LDL particle transcytosis, endothelial cells activation, and induced protherogenic molecule CD36 and vascular cell adhesion molecule-1 expression [12,38–39]. In this paper, we conclude that CAV-1 plays a crucial role in inhibiting CypA-mediated ROS production, improving dyslipidemia, maintaining mitochondrial function, and attenuating oxidative stress responses that are vital for cell survival in hypercholesterol-affected renal organs. Cav-1 ameliorates nephrotic damage in a rabbit model of cholesterol-induced hypercholesterolemia.

## Supporting Information

S1 File Datasets of CAV-1 and CypA on immunohistochemical staining in rabbit renal tissue. (XLSX)

S2 File Datasets of lipid accumulation and lipid peroxidation in rabbit renal tissue. (A) Oil Red O staining. (B) MDA levels.
(XLSX)

S3 File Datasets of mitochondrial compensatory action. (A) mtDNA copy number per cell in rabbit renal tissue. (B) PGC-1α and NRF-1 on immunohistochemical staining.
(XLSX)

S4 File The degree of oxidative damage datasets. (A) 8-OHdG IHF staining in rabbit glomerular tissue. (B) mtDNA oxidative damage in rabbit renal tissue.
(XLSX)

S5 File Datasets of antioxidant enzyme expression, SOD2 and catalase on IHC staining. (XLSX)

S6 File Datasets of the mitochondrial function, biogenesis, inflammation and antioxidation by western blotting. (A) The OXPHOS complex (I–V) band intensities. (B) The CAV-1, CyPA, mitochondrial biogenesis markers, and antioxidant enzymes.
(XLSX)

S7 File Datasets of the apoptotic cell death, TUNEL IHF stain in rabbit renal tissue. (XLSX)

S8 File Datesets of Nrf2, Keap1, and GCLC on IHC staining. (XLSX)

S9 File Datasets of Nrf2-Keap1-GCLC pathway by western blotting. (A) Nrf2 experssion, (B) Keap1 expression, and (C) GCLC expression in the total homogenate, cytoplasm and nuclear fractions.
(XLSX)
